# Truncated Pleurocidin Derivative with High Pepsin Hydrolysis Resistance to Combat Multidrug-Resistant Pathogens

**DOI:** 10.3390/pharmaceutics14102025

**Published:** 2022-09-23

**Authors:** Dejuan Wang, Jingru Shi, Chen Chen, Zhiqiang Wang, Yuan Liu

**Affiliations:** 1 College of Veterinary Medicine, Yangzhou University, Yangzhou 225009, China; 2 Jiangsu Co-Innovation Center for Prevention and Control of Important Animal Infectious Diseases and Zoonoses, Yangzhou University, Yangzhou 225009, China; 3 Joint International Research Laboratory of Agriculture and Agri-Product Safety, the Ministry of Education of China, Yangzhou University, Yangzhou 225009, China; 4 Institute of Comparative Medicine, Yangzhou University, Yangzhou 225009, China

**Keywords:** antimicrobial resistance, AMPs, bacteria, Pleurocidin

## Abstract

The global prevalence of antimicrobial resistance calls for the development of novel antimicrobial agents, particularly for these orally available drugs. Structural modifications of the natural antimicrobial peptides (AMPs) provide a straightforward approach to develop potent antimicrobial agents with high specificity and low toxicity. In this study, we truncated 11-amino-acids at the C-terminus of Pleurocidin, an AMP produced by *Pleuronectes americanus*, and obtained four peptide analogues termed GK-1, GK-2, GK-3 and GK-4. Minimum inhibitory concentration (MIC) tests showed that GK-1 obtained by direct truncation of Pleurocidin has no antibacterial activity, while GK-2, GK-3 and GK-4 show considerable antibacterial activity with Pleurocidin. Notably, GK-4 displays rapid bacteriostatic activity, great stability and low hemolysis, as well as enhanced hydrolytic resistance to pepsin treatment. Mechanistic studies showed that GK-4 induces membrane damage by interacting with bacterial membrane-specific components, dissipates bacterial membrane potential and promotes the generation of ROS. SEM and CD analysis further confirmed the ability of GK-4 to resist pepsin hydrolysis, which may be attributed to its stable helicity structure. Collectively, our findings reveal that GK-4 is a potential orally available candidate to treat infections caused by multidrug-resistant pathogens.

## 1. Introduction

The increasing growth and global spread of drug-resistant bacteria have prompted the need of new generation of antimicrobial agents [1]. Antimicrobial peptides (AMPs) are considered to be one of the most promising alternative drugs. Different from conventional antibiotics, bacteria are not easy to develop resistance to AMPs, because the mechanism of action of AMPs is diverse, ranging from membrane damage to interaction with intracellular targets [2]. However, some inherent defects still seriously hinder the progress of its therapeutic application, such as poor stability under physiological salt concentration and serum conditions, high toxicity to mammalian cells and low resistance to protease degradation [3]. The low resistance to pepsin further prevents the oral application of AMPs in clinical practice [4]. Despite the difficulties, effective AMPs that can be taken orally are an important goal of the pharmaceutical industry. However, almost none of the more than 60 approved peptides can be administered orally. One exception is the GLP-1 receptor antagonist semaglutide [5], which is approved for oral treatment for type 2 diabetes in 2019 by Food and Drug Administration (FDA). Oral administration is the preferred mode of administration, because it facilitates self-administration and can be adjusted widely [6]. If there are adverse reactions, this administration can be terminated quickly [7]. Heretofore, many complex chemical synthesis methods have been proposed, such as polymerization, peptide mimicry and the use of expensive unnatural amino acids [8], to improve the protease hydrolytic resistance of AMPs, but at the same time, it significantly increases the manufacturing cost of AMPs, and the high manufacturing cost is an important obstacle for the commercialization of AMPs. Therefore, shortening and reasonable rearrangement of natural amino acids to avoid protease cleavage sites is a promising method [9], which can simultaneously improve the protease hydrolytic resistance of AMPs and reduce their production cost [10]. Of course, the modification of known peptides with excellent activity and low toxicity is more likely to be successful.

Pleurocidin (C_129_H_192_N_36_ MW: 2711.1, hereinafter referred to as Pleu) is a natural polypeptide isolated from the epidermis of American plaice [11]. Studies have shown that Pleu has a broad spectrum of antibacterial activity and can resist a variety of bacteria and fungi. In addition, compared with other natural peptides, Pleu shows lower hemolysis in in vitro toxicity study, and it has shown that Pleu has an inhibitory effect on common cariogenic bacteria and can effectively prevent and control dental caries [12,13]. Therefore, Pleu has a potential therapeutic value. However, its length is 25 amino acids, and its long amino acid sequence increases its production cost, and its intolerance to pepsin further hinders its clinical application. The aim of this study is to create a shorter Pleu-derived peptide, which can maximize its broad-spectrum antibacterial activity and improve its stability and safety.

The mimic peptide tested in this study was designed by truncating 11 amino acids at the C-terminal of Pleu. Firstly, considering that pepsin prefers to hydrolyze peptide bonds containing aromatic amino acids (Phenylalanine, Tryptophan, and Tyrosine) or Leucine at both ends, thus we truncated the amino acid at the C-terminus of Pleu that could not resist pepsin hydrolysis to obtain GK-1. Secondly, given the effects of amino acid composition, cationic charge, amphiphilicity and α-helicity on antimicrobial activity of AMPs, among them, amphiphilicity is considered to be more important for the activity of AMPs, and the introduction of Tryptophan (Trp) and Lysine (Lys) is a strategy for rational peptide design [9]. Thus, the α-helical and amphiphilic properties were improved by replacing Glycine (Gly), which was not conducive to the formation of α-helical with a highly hydrophobic Trp and hydrophilic Lys. Based on these principles, we substituted amino acids of GK-1 to obtain three variants including GK-2, GK-3 and GK-4.

In this study, through a series of evaluations on the antibacterial activity and stability of the modified peptides, we found that peptide GK-4 has the best performance among all the modified peptides. The antibacterial activity and safety of GK-4 are comparable to those of Pleu before truncation. More importantly, it is more resistant to pepsin treatment and has a lower production cost than Pleu. Finally, we explored the possible mechanisms of action accounting for the antibacterial activity of Pleu and GK-4.

## 2. Materials and Methods

### 2.1. Synthesis and Validation of Peptides

The peptides used in this study were synthesized by GL Biochem company (Shanghai, China) by solid-phase peptide synthesis (SPPS), and their precise molecular weight was verified by matrix-assisted laser desorption/ionization time-of-flight mass spectrometry. The purity of the obtained peptides is more than 95%. Helical wheel projection and various chemical parameters such as net charge and hydrophobicity were calculated with https://www.donarmstrong.com/cgi-bin/wheel.pl (accessed on 10 August 2022).

### 2.2. Determination of Antibacterial Activity

#### 2.2.1. Bacterial Strains and Growing Conditions

*S. aureus* ATCC 29213, *E. coli* ATCC 25922, *S. enteritidis* ATCC 13076 and *A. baumannii* ATCC 19609 were obtained from the American Type Culture Collection (ATCC). The remaining strains used in the experiment were all preserved strains in our laboratory. All strains were cultured in nutrient broth overnight to the logarithmic phase at 37 °C prior to each experiment.

#### 2.2.2. Minimum Inhibitory Concentrations (MICs) Determination

The MICs of all drugs were determined by micro-broth dilution method according to CLSI 2018 guidelines [14]. Briefly, a single colony of the bacterial strain was transferred to 1 mL of Mueller Hilton broth (MHB) and overnight at 37 °C and then 10 μL were cultured in a new 1 mL medium for 4 to 6 h, and then the bacterial culture at the logarithmic phase was diluted to 1 × 10^6^ CFUs/mL. The assay was performed by adding 100 μL of each peptide solution at various concentrations (0.25 to 128 μg/mL) and 100 μL of diluted bacterial culture to different wells of a 96-well microtiter plate (Corning, NY, USA). After incubation at 37 °C for 16 to 18 h and then read its MIC value. MIC value is defined as the lowest drug concentration without visible bacteria.

#### 2.2.3. Determination of Inhibition Curves

The monoclonal strains of *E. coli* B2 and MRSA T144 were cultured overnight at 37 °C and then 10 μL were cultured in a new 1 mL medium for 4 to 6 h. The bacterial suspension was diluted 1000 times (~1 × 10^6^ CFUs/mL) and incubated with different concentrations of drugs (0 to 256 μg/mL) at 37 °C for 0.5, 1, 2 and 4 h, respectively. Among them, Tigecycline (Tig) was selected as a validated quality control. At each of the above time points, 20 μL of mixed culture medium was added to 180 μL of normal saline, and then diluted ten times in sequence [15]. The diluent was taken and dropped onto LB agar plate. After overnight culture at 37 °C, the number of colonies was calculated.

### 2.3. Determination of Hemolysis

Fresh sheep red blood cells (Solarbio, Beijing, China) were washed twice with normal saline, and then 8% red blood cell suspension was prepared. Then, the erythrocyte suspension was incubated with continuously diluted peptides at 37 °C for 1 h. Sterilized normal saline and double-distilled water (ddH_2_O) were used as blank control and positive control, respectively [15,16]. Record OD_576_ of supernatant after centrifugation. Calculate the corresponding hemolysis rate. The hemolysis rate is calculated as follows:

Hemolysis rate (%) = [(OD_576 sample_ − OD_576 blank_)/(OD_576 ddH_2_O_ − OD_576 blank_)] ×100% (1)

### 2.4. Determination of Stability

#### 2.4.1. Temperature and pH Stability

Before MIC determination, the peptides were treated at 20, 40, 60, 80 and 100 °C for 1 h, at 121 °C for 15 min, and under different acid-base environments with pH values of 2, 4, 6, 8, 10 and 12 for 1 h. Then, the antibacterial activity of the peptides affected by different temperatures and pH were determined according to the determination steps of MICs test [10].

#### 2.4.2. Ion and Serum Plasma Stability

In MHB medium, Na^+^, K^+^, Mg^2+^ and Ca^2+^ with the final concentrations of 10 mM, fetal bovine serum, plasma or DMEM medium (Hyclone, Logan, UT, USA) with the final concentrations of 10% was added, and then MIC values were measured to evaluate the effects of different cations, serum and plasma on the antibacterial activity of peptides.

#### 2.4.3. Protease Stability

The peptide was incubated with protease (pepsin, trypsin and papain) (Solarbio, Beijing, China) with a final concentration of 1 mg/mL at 37 °C for 1 h, then the mixture of peptide and protease was heated at 100 °C for 30 min, then centrifuged at 13,000× *g* for 30 min, and the supernatant was taken for MIC determination [17].

### 2.5. Verification of Pepsin Tolerance

#### 2.5.1. Circular Dichroism (CD) Analysis

In four different solvents, including 0.01 M PBS (pH = 7.2), 50 µM LPS (Aladdin, Shanghai, China), 50 mM sodium dodecyl sulfate (SDS) (Aladdin, Shanghai, China) and 50% trifluoroethanol (TFEA) (Aladdin, Shanghai, China) [18,19,20], the secondary structure changes of GK-4 and Pleu before and after 1 mg/mL pepsin treatment at 37 °C for 1 h were measured by J-810 polarization spectrometer (Jasco, Tokyo, Japan), and the peptides concentrations were fixed at 100 µg/mL. Among them, the peptides treated with pepsin need to be heated at 100 °C for 30 min, 13,000× *g* centrifuged for 30 min, and the supernatant was taken for CD spectrum determination to eliminate the influence of pepsin on the experimental results.

#### 2.5.2. Scanning Electron Microscope

The monoclonal antibody of *E. coli* B2 was cultured overnight in 30 mL MHB medium, and then the bacteria were washed with normal saline. After that, the bacteria were co-incubated with GK-4 and Pleu before and after treatment with 10 mg/mL pepsin for 1 h, respectively, and the drug concentration remained the same (10–fold MIC). After that, the bacteria in different groups were washed three times with normal saline, fixed with 2.5% glutaraldehyde (Solarbio, Beijing, China) and overnight at 4 °C. The next day, the bacteria were dehydrated with different concentrations of alcohol (30%, 50%, 70%, 90% and 100%). Finally, after the bacteria were dried, plated and adhered, and the samples were observed by GeminiSEM 300 (Carl Zeiss, jena, Germany).

### 2.6. Study on Antibacterial Mechanism

#### 2.6.1. Cell Membrane Integrity Test

The integrity of the bacterial cell membrane was detected by fluorescent dye propidium iodide (PI) (Beyotime, Shanghai, China) [21]. The cell suspensions of *E. coli* B2 and MRSA T144 (OD_600_ = 0.5) were incubated with PI (0.5 µM) in the dark for 30 min, respectively, and then the labeled cells were treated with increased concentrations (0 to 32–fold MIC) of peptides. After incubation for 1 h, the fluorescence intensity was measured by an Infinite M200 Microplate reader (Tecan, Männedorf, Switzerland) (λex/λem = 535/615 nm).

#### 2.6.2. Measurement of Plasma Membrane Potential

The plasma membrane potential of bacterial cells was measured by 3′,3′-dipropylthiacarbocyanine iodide (DiSC_3_(5)) (Aladdin, Shanghai, China) [22]. The cell suspensions of *E. coli* B2 and MRSA T144 (OD_600_ = 0.5) were incubated with DiSC_3_(5) (0.5 µM) in the dark for 30 min, respectively, and then the labeled cells were treated with increased concentrations (0 to 32–fold MIC) of peptides. After incubation for 1 h, the fluorescence intensity (λex/λem = 622/670 nm) was measured by an Infinite M200 Microplate reader (Tecan, Männedorf, Switzerland).

#### 2.6.3. ROS Determination

The production level of bacterial ROS was measured by 2′,7′-dichlorodihydrofluorescein diacetate (DCFH-DA, Beyotime, Jiangsu, China) [23]. The cell suspensions of *E. coli* B2 and MRSA T144 (OD_600_ = 0.5) were incubated with DCFH-DA (10 µM) in the dark for 30 min, respectively, and then the excess fluorescent probes that did not enter the cells were washed away with PBS. The labeled cells were then treated with increased concentrations (0 to 32–fold MIC) of peptides. After incubation for 1 h, the fluorescence intensity was measured by Infinite M200 Microplate reader (Tecan, Männedorf, Switzerland) (λex/λem = 488/525 nm).

Next, to assess the role of ROS production in the antibacterial activity of GK-4 and Pleu, increasing concentrations of N-acetylcysteine (NAC) (Sigma Aldrich, Saint Louis, MO, USA) from 0 to 5 mM were added to the culture medium, followed by MICs test.

#### 2.6.4. LPS and Phospholipid Inhibition Test

The checkerboard microdilution method was used to evaluate the effects of LPS and phospholipids (Sigma Aldrich, Saint Louis, MO, USA) on the antibacterial activity of peptides [24]. Phospholipids include phosphatidylcholine (PC), phosphatidylethanolamine (PE), phosphatidylglycerol (PG) and cardiolipin (CL) [25]. In short, the effect of different concentrations of LPS (0 to 128 µg/mL) and phospholipids (0 to 16 µg/mL) on the antimicrobial activity of peptides was determined by the fold change of MIC.

## 3. Results

### 3.1. Physicochemical Properties of Peptides

**Figure 1:**
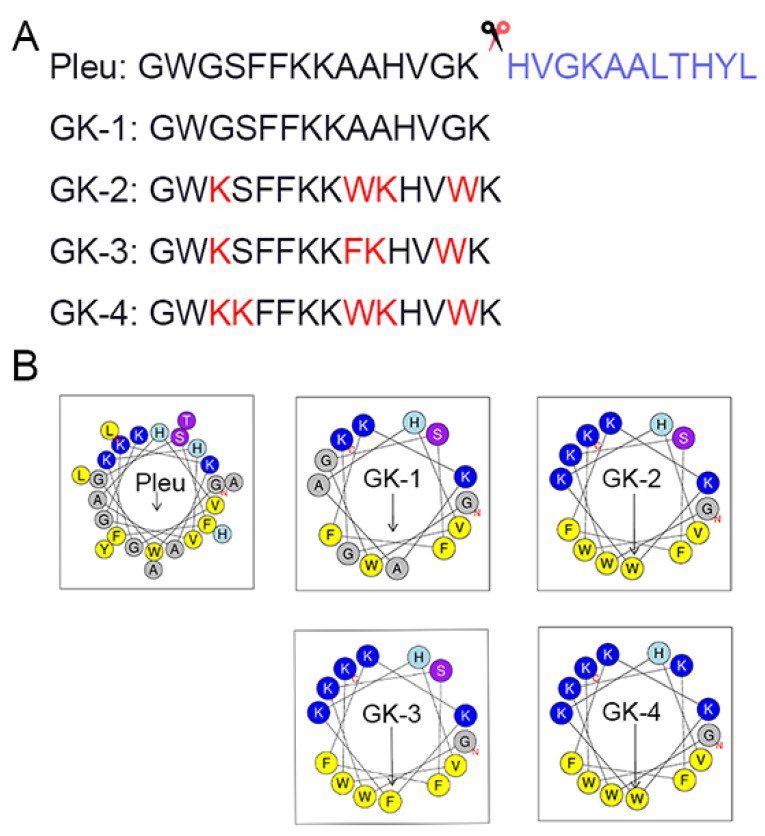
Design and characterization of Pleu and its modified peptides. (**A**) The amino acid sequences of different peptide mutants. (**B**) Helical wheel projections of peptides. Amino acids in blue are positively charged, while in yellow are hydrophobic.

As shown in Figure 1A, GK-1 was obtained by truncating 11 amino acids at the C-terminal of Pleu. Next, some amino acids were substituted for GK-1 to obtain GK-2, GK-3 and GK-4. The number of amino acids of the three modified peptides was consistent with that of GK-1, which all had 14 amino acids. According to the helical wheel diagram in Figure 1B, compared with the GK-1 obtained by direct truncation, the amphiphilicity of the modified peptides was significantly enhanced. After that, we conducted an online analysis on the physicochemical properties of Pleu and its modified peptides and found that the molecular weights of all the modified peptides decreased relative to Pleu. Pleu and its modified peptides were cationic peptides. The net charge of GK-1 was decreased relative to Pleu, but that of its three modified peptides was increased compared to Pleu. In addition, the lipid solubility index of all modified peptides was reduced. The lipid solubility index of GK-1 (35.00) was about two times lower than that of Pleu (70.40). Compared with GK-1, the lipid solubility index of the three modified peptides was decreased to 20.71 (Table 1).

### 3.2. Broad-Spectrum Antibacterial Activity of Peptides

**Figure 2:**
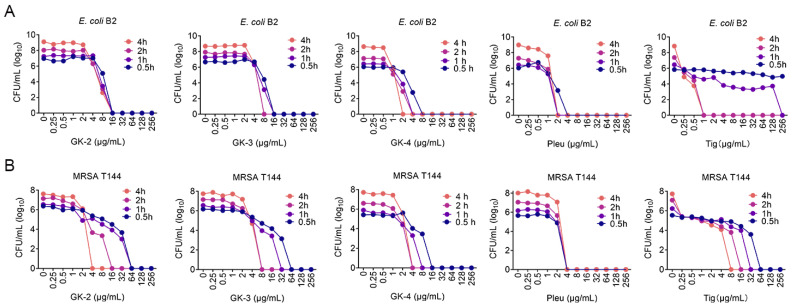
The inhibition curves of Pleu and its modified peptides against *E. coli* B2 (**A**) and MRSA T144 (**B**). The mixtures of varying concentrations of drugs (0 to 256 µg/mL) and bacterial suspension were incubated at 37 °C for 0.5, 1, 2, and 4 h, respectively. Afterwards, 10–fold serially suspensions were plated on LB plates and incubated overnight, and the corresponding CFUs were counted and calculated. Tigecycline (Tig) was selected as a validated quality control. Experiments were conducted with biological replicates and data were presented as mean ± s.d.

Next, we compared the antibacterial activity of Pleu and its four modified peptides against a panel of bacterial strains, including clinically important pathogens. MICs analysis showed that GK-1 obtained by direct truncation of Pleu almost lost its antibacterial ability, while GK-2, GK-3 and GK-4 displayed good antibacterial activity against both Gram-negative and -positive bacteria carrying different drug resistance genes, including methicillin-resistant *Staphylococcus aureus* (MRSA), NDM-positive *E. coli*, *mcr/tet*(X)*/tmexCD1-torJ1*-carrying pathogens (Table 2). After that, we carried out the inhibition curves to assess whether these peptides have obvious bactericidal activity in a short time. As shown in Figure 2, Pleu and its modified peptides had a rapid sterilization effect on both MRSA T144 and *E. coli* B2 and showed obvious time and concentration dependence. Among all the modified peptides, GK-4 exhibited the best antibacterial effect on both MRSA T144 and *E. coli* B2, which is more obvious in the inhibition curve of MRSA T144.

### 3.3. High Stability and Safety of Peptides

The stability of AMPs is an important prerequisite for their effectiveness in vivo [26,27]. Thus, we evaluated the changes of antibacterial activity of Pleu and its modified peptides at different temperatures (40 to 121 °C) and pH (2 to 12). As a consequence, we found that Pleu and its modified peptides still maintained stable antibacterial activity after being treated at different temperatures and pH for 1 h (Table 3). In addition, we assessed the effects of different ions such as Na^+^, K^+^, Mg^2+^ and Ca^2+^ on the antibacterial activity of four peptides. We found that monovalent cations had little effect on the antibacterial activity of peptides, while divalent cations greatly weakened the activity of Pleu and its modified peptides (Table 3). Furthermore, the media containing 10% fetal bovine serum, plasma and DMEM were used to simulate the matrix environment in vivo [27]. The results showed that these three substances would not weaken the antibacterial activity of the compounds we tested. Finally, we assessed the protease hydrolytic resistance of Pleu and its modified peptides. We found that trypsin and papain almost completely destroyed their antibacterial activity, while GK-4 showed unique advantages over the other two modified peptides for its higher hydrolytic resistance to pepsin (Table 3). The good anti-pepsin hydrolytic ability of GK-4 provides a basis for its further oral application. These results imply that GK-4 has the best stability in vitro among all the modified peptides. In view of this characteristic, we selected GK-4 as a lead for further research.

**Figure 3:**
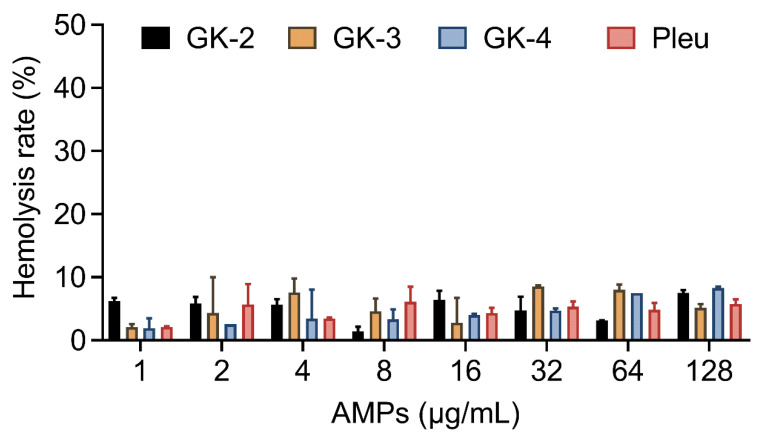
Hemolytic activity of Pleu and its modified peptides against sheep red blood cells (RBCs). Sterilized PBS and ddH_2_O were used as negative control and positive control, respectively.

Safety is the key factor that hinders the entry of AMPs into the clinical application [28,29]. Therefore, we measured the hemolytic rate of Pleu and its modified peptides. The results showed that the hemolysis rate of GK-4 with high pepsin hydrolysis resistance on sheep erythrocytes in the concentration range of 1 to 128 μg/mL was less than 10% (Figure 3).

### 3.4. GK-4 Exerts Antimicrobial Activity by Interacting with Membrane Components

**Figure 4:**
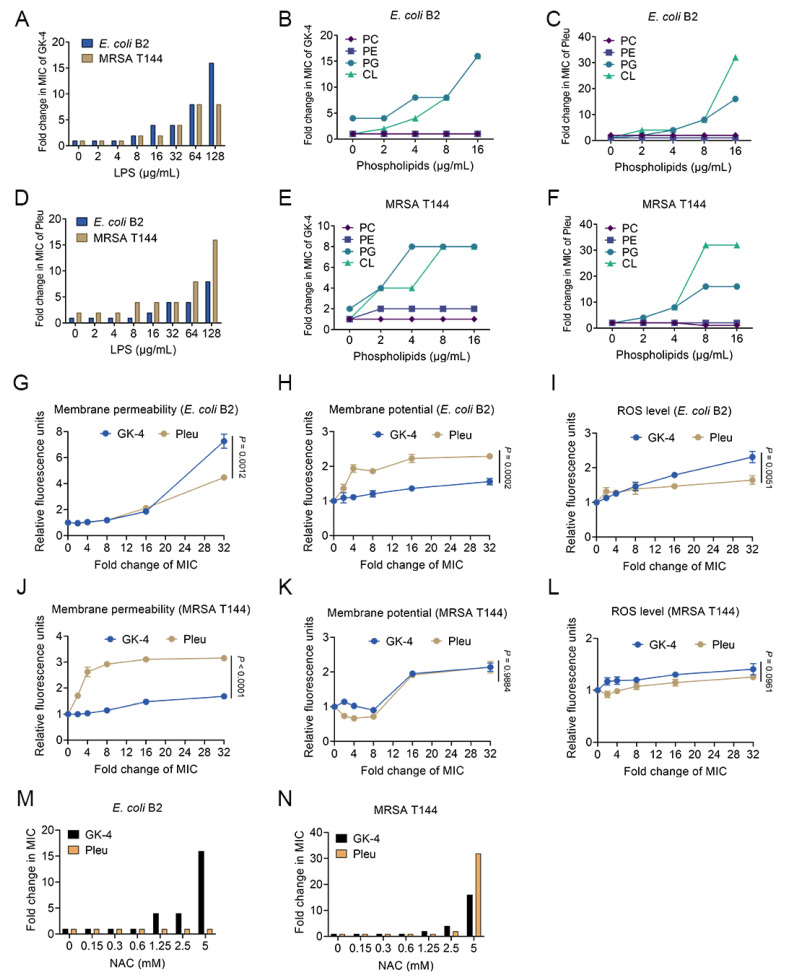
Antibacterial mechanisms of GK-4. (**A**,**D**) Exogenous addition of LPS deriving from *E. coli* O111:B4 impaired the antibacterial activity of GK-4 and Pleu against *E. coli* B2 and MRSA T144 in a dose-dependent manner. (**B**,**E**) Increased MICs of GK-4 against *E. coli* B2 and MRSA T144 in the presence of PC, PE, PG and CL. The 96-well plates were incubated at 37 °C for 16 to 18 h for MIC analysis. (**C**,**F**) Increased MICs of Pleu against *E. coli* B2 and MRSA T144 in the presence of PC, PE, PG and CL. The 96-well plates were incubated at 37 °C for 16 to 18 h for MIC analysis. (**G**,**J**) GK-4 disrupted the whole cell membrane permeability of *E. coli* B2 and MRSA T144, which were assessed by propidium iodide (PI, excitation 535 nm and emission 615 nm). Bacterial cells were incubated with fluorescent probes at 37 °C for 30 min in dark, then different concentrations of GK-4 (0 to 32 × MIC) were added and incubated at 37 °C for 1 h. (**H**,**K**) GK-4 dissipated the membrane potential of *E. coli* B2 and MRSA T144, determined by monitoring the fluorescence intensity of 3,3′-dipropylthiadicarbocyanine iodide (DiSC_3_(5), excitation at 622 nm and emission at 670 nm). (**I**,**L**) GK-4 triggered the production of ROS in *E. coli* B2 and MRSA T144. The production of ROS of *E. coli* B2 and MRSA T144 was determined by 2′,7′-dichlorodihydrofluorescein diacetate (DCFH-DA, excitation at 488 nm and emission at 525 nm). (**M**,**N**) Effects of NAC supplementation on the antibacterial activity of GK-4 and Pleu against *E. coli* B2 and MRSA T144. Data in (**A**–**F**,**M**,**N**) represent two biological replicates. All experiments in G-L were performed as three biologically independent experiments, and the mean ± s.d. is shown. *p* values were determined using an unpaired, two-tailed Student’s *t*-test.

After showing that GK-4 has strong antibacterial activity, we tried to further clarify its antibacterial mechanisms. As an important protective barrier for Gram-positive and Gram-negative bacteria, the bacterial cell membrane is usually the target of most AMPs [30,31,32]. In addition to the outer membrane, the cytoplasmic membrane is an important barrier in both Gram-positive and Gram-negative bacteria, PG, CL and PE are the main components of the cytoplasmic membrane, and PC mainly exists in eukaryotic cells. To study whether GK-4 can target these membrane components, different concentrations of exogenous LPS, PG, CL, PE and PC were added into MICs analysis. The results showed that exogenous LPS significantly reduced the antibacterial activity of GK-4 and Pleu against MRSA T144 and *E. coli* B2 (Figure 4A,D), indicating that GK-4 and Pleu had a close interaction with LPS. In addition to LPS, the exogenous addition of PG and CL also significantly weakened the antibacterial activity of GK-4. By contrast, the supplementation of PC and PE did not affect their antibacterial activity (Figure 4B,E). We can also acquire similar results through Figure 4C,F, that is, Pleu can also target PG and CL on the cytoplasmic membrane. The above results show that GK-4 can selectively interact with relevant components in bacterial cell membranes.

Considering that GK-4 could interact with bacterial membrane-specific components, we next evaluated the effect of GK-4 on bacterial membrane permeability using the fluorescent probe propidium iodide (PI). Accordingly, PI cannot pass through the living cell membrane, but it can pass through the damaged cell membrane and bind with DNA to emit red fluorescence [33]. As shown in Figure 4G,J, GK-4 significantly increased the PI fluorescence intensity in both *E. coli* B2 and MRSA T144 in a dose-dependent manner, indicating that it resulted in obvious damage to the bacterial cell membrane, and the membrane damage caused by GK-4 to *E. coli* B2 was more obvious than Pleu at the same 32–fold MIC of drug concentration. These results demonstrate that GK-4 acts by enhancing bacterial membrane permeability.

### 3.5. GK-4 Dissipates Bacterial Membrane Potential and Promotes the Generation of ROS

Membrane potential (Δψ) is one of the important components of bacterial proton motive force (PMF) [34]. It is involved in maintaining the dynamic balance of bacterial proton dynamics and is essential for bacterial survival. We next evaluated the effect of GK-4 peptide on membrane potential using the fluorescent dye DiSC_3_(5) [35], as it accumulates in the cytoplasmic membrane in response to the Δψ component of bacteria. When Δψ is destroyed, the probe will be released into the extracellular environment, resulting in fluorescence enhancement. Our results showed that the fluorescence intensity of DiSC_3_(5) in MRSA T144 and *E. coli* B2 was significantly increased in a dose-dependent manner after treatment with increased concentrations of GK-4 peptide (Figure 4H,K), suggesting that GK-4 disrupted Δψ formation of the proton motive force (PMF).

As a strong oxidant, reactive oxygen species (ROS) can cause the rapid oxidation of various biological macromolecules in cells, which is very harmful to cells [36]. Considering the good antibacterial activity of GK-4, we next used DCFH-DA to measure the level of ROS in bacteria applied by GK-4. We found that GK-4 triggered the generation of ROS in a concentration-dependent manner (Figure 4I,L). Moreover, at the same 32–fold MIC of drug concentration, the increase of ROS levels in bacteria by GK-4 was higher than Pleu, especially in *E. coli* B2. To verify the role of ROS production in the antibacterial activity of GK-4, we tested the effect of ROS scavenger NAC on the MICs of GK-4 [37,38]. We found that when the concentration of NAC was 5 mM, the antibacterial activity of GK-4 against *E. coli* B2 and MRSA T144 was decreased significantly. Interestingly, at the same concentration, the addition of NAC did not affect the activity of Pleu on *E. coli* B2, but significantly abolished the activity of Pleu on MRSA T144 (Figure 4M,N), suggesting that GK-4 and Pleu may have different antibacterial mechanisms against Gram-positive and Gram-negative bacteria. Together, these results show that the production of ROS is the key factor accounting for the antibacterial activity of GK-4.

### 3.6. GK-4 Has High Pepsin Hydrolytic Resistance Relative to Pleu

**Figure 5:**
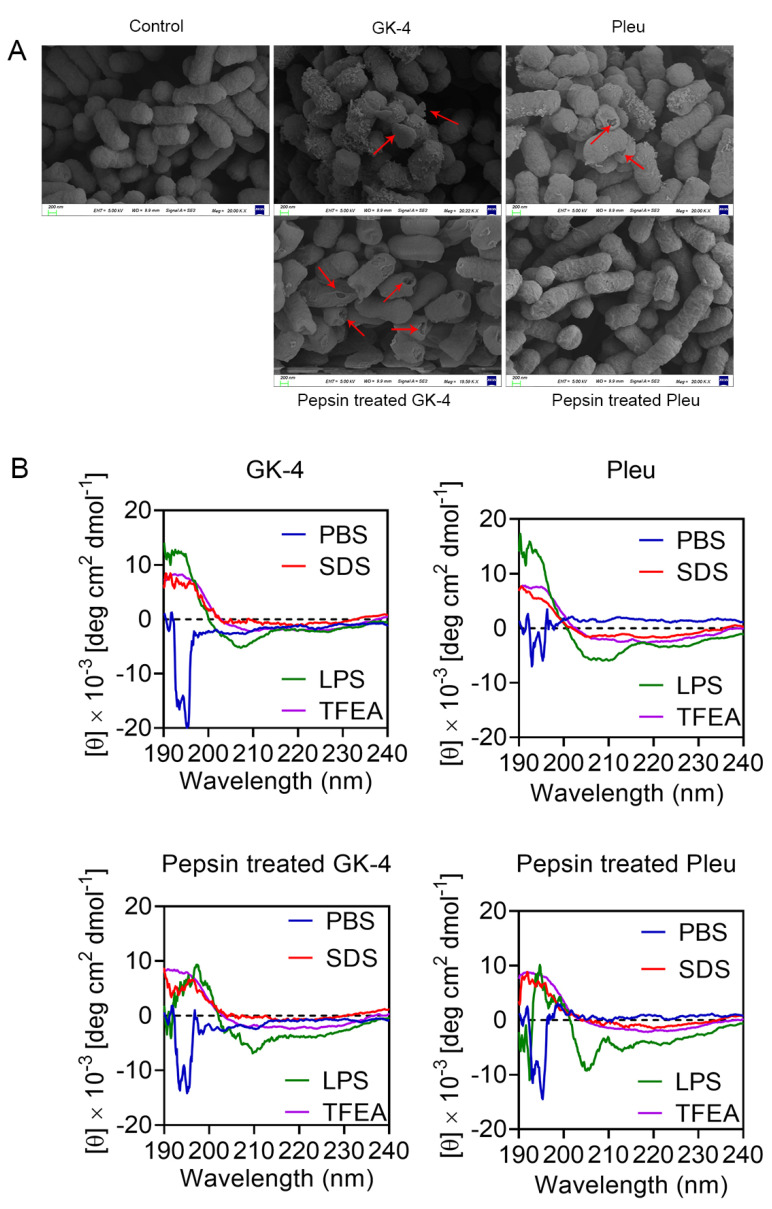
(**A**) SEM images of *E. coli* B2 exposed to GK-4 and Pleu, or GK-4 and Pleu treated with 10 mg/mL pepsin, and the peptide concentrations were fixed at 20 µg/mL (10–fold MIC). All pepsin treated peptides were heated at 100 °C for 30 min and centrifuged at 130,00× *g* for 30 min. Then the supernatant was taken to eliminate the influence of pepsin on subsequent tests. (**B**) Circular dichroism (CD) spectra of GK-4 and Pleu, or GK-4 and Pleu treated with 1 mg/mL pepsin in various solutions. PBS (10 mM, pH = 7.4), lipopolysaccharide (LPS) (50 µM), sodium dodecyl sulfate (SDS) (50 mM), and 50% TFEA were used. The values from three scans were averaged per sample, and the peptide concentrations were fixed at 100 µg/mL. All pepsin treated peptides were heated at 100 °C for 30 min and centrifuged at 13,000× *g* for 30 min. Then the supernatant was taken to eliminate the influence of pepsin on subsequent tests.

The poor resistance to gastric protein has always been an insurmountable obstacle in the clinical application of AMPs [39]. In our above experiments, we found that the antibacterial activity of Pleu was remarkably decreased after pepsin treatment, while the antibacterial activity of the modified peptide GK-4 showed no decrease after pepsin treatment. To further verify this phenomenon, we firstly compared the morphological changes of *E. coli* B2 caused by GK-4 and Pleu before and after treatment with 10 mg/mL pepsin by scanning electron microscopy (SEM) [40,41]. Compared with the control group with complete morphology, pores were formed on the surface of *E. coli* B2 treated with 10–fold MIC concentrations of GK-4 and Pleu, and cell membranes were partially broken or broken into fragments. At the same drug concentration, GK-4 treated with pepsin still caused serious damage to the bacterial cell membrane, while Pleu treated with pepsin almost lost its antibacterial ability and hardly caused damage to the bacterial cell membrane. There was no significant difference between the treated bacteria and the blank control group. These results indicate that GK-4 is more resistant to pepsin treatment than Pleu (Figure 5A).

Furthermore, we determined the secondary structures of GK-4 and Pleu in different solvents by CD analysis. As LPS is an important part of the outer membrane of Gram-negative bacteria, LPS is selected to simulate the bacterial cell environment. SDS and TFEA with the negative charge on the surface were used to simulate the anionic membrane environment and hydrophobic environment of the bacterial cell membrane, respectively. As shown in Figure 5B, GK-4 and Pleu showed obvious disordered structure in PBS solution, while the helix ratio showed an obvious upward trend in 50 µM LPS, 50 mM SDS and 50% TFEA. Based on the secondary structure analysis, the helix ratio of GK-4 and Pleu treated with 1 mg/mL pepsin in 50 mM SDS and 50% TFEA did not change compared with GK-4 and Pleu in the blank control group, but in 50 µM LPS, the helix ratio of GK-4 treated with pepsin was decreased by about 2.6–fold, while that of Pleu treated with pepsin was reduced by about 5.2–fold (Table 4). Therefore, we conclude that the more stable secondary structure may account for the higher pepsin hydrolytic resistance of GK-4 than its parent Pleu.

## 4. Discussion

The emergence, prevalence and rapid spread of antibiotic resistance in humans, animals and environment pose an increasingly serious threat to public health all over the world. In particular, the emergence of new ARGs has greatly weakened our traditional treatment options in the clinical setting, almost producing the dilemma of no available antibiotics. In the era of drug resistance, AMPs have attracted global attention as a new candidate drug for the treatment of infectious diseases due to the limited choice of existing antibiotic regimens [42]. However, high synthesis costs and poor anti-proteolytic ability are urgent challenges that prevent the clinical application of AMPs. To overcome these issues, we truncated a reported AMP-Pleu and obtained the shorter peptide GK-1. The amino acid sequence, fat solubility index, antibacterial spectrum, antibacterial activity and synthesis cost of GK-1 were significantly reduced. The decrease of antibacterial activity of GK-1 may be related to low cationic charge, amphiphilic and α-helicity, which are the key factors affecting the activity of AMPs. It have shown that α-helical peptides, such as membrane lysed AMPs, have obvious hydrophobic and hydrophilic surfaces, which facilitate the insertion of AMPs into bacterial membranes, resulting in enhanced bacterial membrane permeability and membrane damage [43]. Based on this principle, we further optimized the chemical structure of GK-1 through amino acid replacement and obtained three novel peptides denoted as GK-2, GK-3 and GK-4.

In the MICs results, we found that except for GK-1, the other modified peptides had better broad-spectrum antibacterial activity, which may be due to their more obvious amphipathic distribution. In the inhibition curves analysis, we followed the principle that peptides that can inhibit bacteria in a shorter time at the same concentration or at a lower concentration during the same time will have better antimicrobial activity. We found that all the three modified peptides had good antibacterial activity, and GK-4 had the best antibacterial activity among them. Of course, its antibacterial effect was still slightly inferior to Pleu, but compared with Pleu, GK-4 had obvious price advantage, which is important for its clinical application [44]. Stability tests showed that Pleu and its modified peptides had good thermal stability, pH stability, and serum plasma stability. We speculated that the high stability of the peptides may be related to their nonspecific or mixed secondary structures in solution, which was confirmed by following CD detection. However, in the salt ion stability test, we found that monovalent cations had little effect on the antibacterial activity of peptides, while divalent cations greatly weakened the activity of Pleu and its modified peptides. Since divalent cations are stabilizers of the bacterial extracellular membrane [45], we inferred that the antibacterial mechanisms of Pleu and its modified peptides may be related to cell membrane damage.

Importantly, in the evaluation of protease stability, we found that only GK-4 peptide was resistant to pepsin treatment. Then, the high pepsin hydrolysis resistance of GK-4 was verified by SEM and CD analysis. The results obtained from our two experiments are highly consistent in that GK-4 was more resistant to pepsin hydrolysis than Pleu. Among them, the determination of CD analysis also provided some theoretical support for the high pepsin hydrolysis resistance of GK-4, that is, GK-4 had a more stable helical structure than Pleu in LPS environment. As previous study have shown inducing rather than forming a stable secondary structure is conducive to the bactericidal effect of AMPs [46], the existence of this merit is extremely favorable for the oral utilization of GK-4.

There are three main mechanisms by which a short α-helical AMP acts on bacterial cell membranes [47]: (1) “barrel-stave” model—AMPs penetrate vertically into the membrane and form oligomeric pore complexes with the hydrophilic region of the peptide facing the pore cavity and the hydrophobic region interacting with the lipid bilayer; (2) “carpet” model—AMPs act at high enough concentrations to accumulate in parallel with lipid bilayers, covering local areas in a carpet-like manner. This results in a detergent-like activity, leading to membrane rupture and lipid formation of lipid-peptide micelles; (3) “toroidal-pore” model—conformational rearrangements and noncovalent dimerization of AMPs occur in the membrane environment, resulting in toroidal pore formation. In this case, the hydrophilic region of the peptide is coordinated with the phospholipid head group, while the hydrophobic region is associated with the lipid core. As for the antibacterial mechanisms of GK-4, we found that GK-4 is a membrane-active peptide antibiotic. This result was directly demonstrated by SEM. GK-4 did cause obvious damage to the bacterial cell membrane. This is also the main mechanism by which most AMPs exert an antibacterial effect. In our study, we further revealed that GK-4 exerted antibacterial activity by interacting with LPS, PG and CL. Considering that these targets have negatively charged head groups, we reasoned that the electrostatic force between positively charged GK-4 and these targets may be the reason for their membrane interaction. PC and PE are abundant in eukaryotic cells. For example, PE accounts for 25% of the human erythrocyte membrane [48], and the content of PC in brain nerve cells accounts for about 17–20% of its mass. The existence of these two components will hardly affect the antibacterial activity of GK-4, that is, GK-4 can selectively bind to bacterial membrane-related components, which is beneficial for its future clinical application. In addition, GK-4 can also cause the loss of bacterial membrane potential and the production of ROS. This is also consistent with a series of previous research results [49,50,51], that is, the loss of membrane potential and the production of ROS are the key factors for the bactericidal activity of many antibiotics.

## 5. Conclusions

To sum up, we identify a novel tetradeceptide termed GK-4 via chemical modification of Pleu, which has good selectivity for bacteria and great stability to drastic pH value, temperature change and high salt ion concentration. In addition, the composition and arrangement of amino acids make the cost of GK-4 much lower and remarkably enhance the hydrolytic resistance to pepsin. Collectively, our findings suggest that GK-4 is a promising candidate drug that can be used orally and has great therapeutic potential against the increasing multidrug-resistant pathogens.

## Figures and Tables

**Table 1:** Key physicochemical parameters of amphipathic peptides.

AMPs	Formula	MW	N	H	FSI	GRAVY	pI	Purity
Pleu	C_129_H_192_N_36_O_29_	2711.17	4	0.421	70.40	−0.068	10.18	96.68%
GK-1	C_73_H_106_N_20_O_16_	1519.77	3	0.342	35.00	−0.314	10.30	95.59%
GK-2	C_97_H_134_N_24_O_16_	1892.28	5	0.478	20.71	−1.200	10.60	95.35%
GK-3	C_95_H_133_N_23_O_16_	1853.25	5	0.445	20.71	−0.936	10.60	95.58%
GK-4	C_100_H_141_N_25_O_15_	1933.38	6	0.410	20.71	−1.421	10.70	95.52%

MW: Molecular Weight; N: Net Charges; H: Hydrophobic; GRAVY: the grand average of hydropathy; FSI: Fat-soluble Index.

**Table 2:** Antibacterial activity of Pleu and its modified peptides against a panel of MDR pathogenic bacteria (MIC, µg/mL).

Organism and Genotype	GK-1	GK-2	GK-3	GK-4	Pleu	Tig
Gram-positive bacteria						
*S. aureus* ATCC 29213	>128	4	4	4	4	0.125
MRSA T144	>128	8	8	4	2	0.25
MRSA 1530	>128	32	16	16	8	1
*S. aureus* 215 (*cfr* + LZD^R^)	>128	4	4	8	2	0.25
*S. aureus* G16 (RIF^R^)	>128	8	8	8	2	<0.0625
*E. faecalis* A4 (VRE, VanA)	>128	16	32	16	8	<0.0625
Gram-negative bacteria						
*E. coli* ATCC 25922	>128	8	8	8	1	0.125
*E. coli* B2 (*bla*_NDM-5_ + *mcr-1*)	>128	2	4	2	2	2
*E. coli* C3 (*bla*_NDM-1_)	>128	2	4	2	1	2
*E. coli* G6 (*bla*_NDM-5_)	>128	2	4	4	1	2
*E. coli* G92 (*mcr-1*)	>128	2	2	4	1	4
*E. coli* CP131 (*mcr-3*)	>128	2	2	2	1	2
*E. coli* B3-1 (*tet*(X4))	>128	4	2	4	1	32
*E. coli* 1F28 (*tet*(X4))	>128	16	16	16	4	16
*S. enteritidis* ATCC 13076	>128	4	4	4	1	0.125
*A. baumannii* ATCC 19609	>128	8	4	4	2	0.25
*P. aeruginosa* PA14	>128	16	64	16	1	<0.0625
*P. aeruginosa* (VIM + *tmexCD1-torJ1*)	>128	16	16	16	4	<0.0625
*P. cibarius* HNCF44W (*bla*_NDM-1_+ *tet*(X6))	>128	8	8	4	2	64

Tig, tigecycline; LZD, linezolid; RIF, rifampicin.

**Table 3:** Thermal, pH, salts and protease stability of Pleu and its modified peptides against MRSA T144 and *E. coli* B2 (MIC, µg/mL).

Treatment	MRSA T144				*E. coli* B2			
	GK-2	GK-3	GK-4	Pleu	GK-2	GK-3	GK-4	Pleu
Control	8	8	4	2	2	4	2	2
Temperature								
40 °C	2	4	2	2	2	2	2	1
60 °C	4	2	2	1	4	2	2	1
80 °C	2	4	2	2	2	2	2	1
100 °C	4	8	4	4	2	4	2	2
121 °C	4	8	4	8	2	4	2	2
pH								
2	4	4	4	4	2	4	1	1
4	4	4	4	4	4	2	1	1
6	4	4	4	2	2	2	2	1
8	4	4	4	4	2	2	1	1
10	4	4	4	4	2	2	1	1
12	4	4	4	4	2	2	2	1
Salts (10 mM)								
Na^+^	2	2	2	2	2	2	2	1
K^+^	4	4	4	2	2	2	2	1
Mg^2+^	4	8	8	8	32	32	32	128
Ca^2+^	>128	>128	>128	>128	>128	>128	>128	>128
Protease (1 mg/mL)								
Pepsin	64	64	8	64	128	64	2	128
Trypsin	>128	>128	>128	>128	>128	>128	>128	>128
Papain	>128	>128	>128	>128	>128	>128	>128	>128
Serum (10%)	8	8	8	4	4	8	4	1
Plasma (10%)	4	4	4	2	0.5	0.5	2	<0.25
DMEM (10%)	4	4	2	2	4	4	4	4

**Table 4:** Secondary structures of GK-4 and Pleu before and after 1 mg/mL pepsin treatment.

Secondary	GK-4				Pepsin Treated GK-4				Pleu				Pepsin Treated Pleu			
Structure	PBS	LPS	SDS	TFEA	PBS	LPS	SDS	TFEA	PBS	LPS	SDS	TFEA	PBS	LPS	SDS	TFEA
Helix	6.6	74.3	100	100	5.6	28.5	100	100	0	68.6	90	100	0	13.2	100	100
Beta	0	0	0	0	0	32.1	0	0	0	0	0	0	0	53.1	0	0
Turn	36.6	0	0	0	34.4	21.3	0	0	100	0	0	0	63.4	11.8	0	0
Random	56.8	25.7	0	0	60	18.1	0	0	0	31.4	10	0	36.6	21.9	0	0
Total	100	100	100	100	100	100	100	100	100	100	100	100	100	100	100	100

## Data Availability

Not applicable.
